# How does Family Socioeconomic Status influence Children's Cardinality Principle? The chain mediation roles of Approximate Number System and Receptive Vocabulary Skills

**DOI:** 10.3389/fpsyg.2025.1530885

**Published:** 2025-07-09

**Authors:** Huanhuan Li, Yifang Wang, Bingyu Duan, Qiaoke Kuang, Xinxin Guo, Enwei Xu

**Affiliations:** ^1^College of Educational Science, Xinjiang Normal University, Urumqi, China; ^2^Shanghai Institute of Early Childhood Education, Shanghai Normal University, Shanghai, China

**Keywords:** Family Socioeconomic Status, Cardinality Principle, chain mediation, Approximate Number System, Receptive Vocabulary Skills

## Abstract

**Background:**

Family Socioeconomic Status, the Approximate Number System, and the Receptive Vocabulary Skills have been identified as key factors influencing the development of children's Cardinality Principle. However, the relationships among these variables and their underlying mechanisms remain unclear. This study aims to investigate how the Family Socioeconomic Status (FES) impacts children's understanding of the Cardinality Principle, with a focus on the mediating roles of the Approximate Number System (ANS) and the Receptive Vocabulary Skills (RVS).

**Methods:**

A cross-sectional design was employed, involving 110 children (55 boys, 55 girls; Mean age = 67.53 months, *SD* = 7.415 months) and their parents. Parents provided information on the Family Socioeconomic Status (income, education level, and occupation). Children's performance on the Approximate Number System, the Receptive Vocabulary Skills, and the Cardinality Principle (CP) tasks was assessed using the Panamath software, the Peabody Picture Vocabulary Test - Revised (PPVT- R), the “How Many” task, and the “Give-N” task. Data were analyzed using SPSS 29.0 and PROCESS v4.1 (Model 6).

**Results:**

Correlation analyses revealed significant positive associations between children's Cardinality Principle, Family Socioeconomic Status, Approximate Number System, and Receptive Vocabulary Skills. After controlling for child gender and age, mediation analysis indicated that the Family Socioeconomic Status had a direct positive effect on children's Cardinality Principle. The Approximate Number System and the Receptive Vocabulary Skills each partially mediated the relationship between Family Socioeconomic Status and Cardinality Principle. Additionally, the Approximate Number System and the Receptive Vocabulary Skills together acted as chain mediators in the Family Socioeconomic Status- Cardinality Principle relationship.

**Conclusions:**

This study highlights that the Family Socioeconomic Status indirectly influences the development of the Cardinality Principle through the Approximate Number System and Receptive Vocabulary Skills, underscoring the complex interactions between the Family Socioeconomic Status, the Approximate Number System, and Receptive Vocabulary Skills in the development of children's mathematical abilities. The findings suggest that early educational interventions should consider both cognitive and language development alongside socioeconomic factors to better support children's mathematical learning.

## 1 Introduction

It is widely accepted that children progress through a systematic and staged process in learning the cardinal meanings of numbers from “one” to “four.” Initially, children may be considered pre-numerical, gradually developing into one-number, two-number, and three-number recognizers. For some, a fourth-number recognition level may also be achieved (Condry and Spelke, 2008). At this stage, children's understanding of the cardinal meaning of numbers is limited by a small capacity system. However, when children grasp the Cardinality Principle (CP), they experience a cognitive leap (Gelman and Gallistel, 1978), realizing that the final number word spoken in a counting sequence represents the total quantity of the set (Sarnecka and Lee, 2009; Jordan et al., 2022). Mastery of the CP is a crucial milestone in early numerical development (Orrantia et al., 2022), laying the foundation for mathematical cognition, and is crucial for the development of logical thinking, problem-solving, and abstract reasoning skills, which can significantly impact future academic achievement (Paliwal and Baroody, 2020; Chen, 2024). Understanding CP marks a critical transition from intuitive number sense to abstract number comprehension (Elgavi and Hamo, 2024), providing a key basis for the development of advanced mathematical abilities (Orrantia et al., 2022).

Given the central role of the CP in early mathematics, researchers have increasingly focused on factors that may influence its development, including the Family Socioeconomic Status (SES), the Approximate Number System (ANS), and the Receptive Vocabulary Skills (RVS). While these factors are generally acknowledged as important, the specific mechanisms linking them to CP development remain unclear. Some studies highlight the significant role of SES in children's CP development, suggesting that economic and social resources may directly influence children's understanding of the CP (Ramani and Siegler, 2008; Jordan et al., 2009). Other research posits that the impact of SES on CP may be mediated by children's cognitive abilities, indicating that individual differences may play a significant role (Duncan et al., 2017; Bachman et al., 2022). Additionally, both ANS and RVS are considered key components of cognitive development and have been shown to influence CP acquisition, though there is no consensus on their specific roles or the extent of their effects (Carey et al., 2017; vanMarle et al., 2018; Negen and Sarnecka, 2012; Chow and Ekholm, 2019).

Despite the growing body of literature on these factors, research exploring their interrelationships and the mechanisms underlying their effects is still limited. In particular, studies examining the role of ANS and RVS as potential mediators, and whether they might collectively exert a chain mediation effect, remain scarce. To address this gap, the present study proposes a chain mediation model to explore both the direct and indirect relationships between SES and children's CP development, testing whether ANS and RVS act as mediators and whether they jointly mediate the SES-CP link. This research aims to provide a comprehensive theoretical framework for understanding how socioeconomic factors shape early mathematical cognition through cognitive and linguistic mechanisms.

### 1.1 Family Socioeconomic Status and Children's Cardinality Principle

SES is a composite measure of family economic and social position, commonly assessed through indicators such as parental education level, occupational status, and household income (Li et al., 2020). According to ecological systems theory, children's development is shaped by multi-level influences, including the micro-system (e.g., family), meso-system (e.g., school and community), and macro-system (e.g., societal policies and culture). The closer these systems are to the individual, the greater their impact on development (Bronfenbrenner and Morris, 1998). Among these, SES is a key factor influencing cognitive development, particularly in the realm of mathematics (Fan et al., 2022; Mao, 2022).

The impact of SES on children's mathematical skills is evident even before formal schooling begins (Klibanoff et al., 2006). Differences in economic resources between social classes can significantly affect children's early math learning, as SES is associated with varying access to learning opportunities and social experiences that support mathematical development (Jordan and Levine, 2009). Research by Denton et al. (2002) demonstrated that children from higher SES backgrounds perform significantly better in math during preschool and early elementary years compared to those from lower SES backgrounds. Huang (2006) further suggested that parental education and the material resources provided by the family are related to children's performance on tasks involving the CP, written number-symbol representation, and arithmetic operations. The family stress model proposed by Conger and Donnellan (2007) holds that a lower socioeconomic status is associated with increased economic stress. This pressure can lead to family conflicts, reduce parent-child interaction and decrease academic guidance. Such an environment limits children's opportunities for cognitive enrichment and learning guidance, further hindering their early academic development, including mastering concepts such as CP. More recent studies have reinforced this notion, highlighting that children from higher SES families, who are exposed to frequent math-related activities and abundant resources, outperform their lower SES peers in understanding and applying mathematical concepts and skills (Ramani and Siegler, 2008; Silver and Libertus, 2022). Based on these findings, we propose the following hypothesis:

***Hypothesis 1 (H1)***: SES has a positive predictive effect on the development of children's CP.

### 1.2 The mediating role of the Approximate Number System

The ANS is a cognitive mechanism used by humans and other animals to estimate quantities quickly and intuitively, allowing us to compare two sets of objects' relative quantities without precise counting or the use of symbols (Negen and Sarnecka, 2015; Cochrane et al., 2019). While ANS has traditionally been viewed as an innate cognitive system (Dehaene, 2011; Starr and Brannon, 2015), recent studies have shown that its sensitivity may be influenced by environmental factors, suggesting the system's plasticity (Tosto et al., 2014).

Although SES is a well-established environmental factor that affects children's development, there is limited consensus in the literature regarding the relationship between SES and ANS (McNeil et al., 2011; Purpura and Simms, 2018; Slusser et al., 2019; Wei et al., 2020; Bachman et al., 2022). For example, a longitudinal study by Wei et al. (2020) with 173 children (mean age = 67.25 months, *SD* = 3.67) found no correlation between SES (family income and parental education level) and performance on ANS tasks (e.g., Dot Comparison, Number Line Estimation). In contrast, Bachman et al. (2022) reported a significant positive correlation between SES and ANS accuracy in a community-based longitudinal study involving 178 children aged 4 years and their parents.

Numerous studies have emphasized the importance of ANS in the early development of mathematical abilities (Libertus et al., 2011; Bonny and Lourenco, 2013; Vogel and De Smedt, 2021). Cognitive neuroscience research has identified the intraparietal sulcus as a key brain region involved in numerical cognition, suggesting a link between ANS and CP (Dehaene et al., 1996). Despite some opposing views (Libertus et al., 2014; Carey et al., 2017; Schröder et al., 2022), empirical studies largely support the role of ANS in children's mastery of early mathematical concepts, including CP (Libertus et al., 2011; Inglis and Gilmore, 2014; Fuhs et al., 2016; vanMarle et al., 2018). Children with higher ANS sensitivity are more likely to grasp symbolic number knowledge, including cardinality and arithmetic knowledge (Chu et al., 2016; Geary et al., 2013; van Marle et al., 2014).

Given the relationships between SES, ANS, and CP, we hypothesize that ANS may mediate the effect of SES on CP development. Although limited research has directly examined this hypothesis, exploring this possibility will deepen our understanding of how environmental factors influence mathematical development through ANS.

***Hypothesis 2 (H2)***: ANS partially mediates the relationship between SES and children's CP.

### 1.3 The mediating role of Receptive Vocabulary Skills

The development of early mathematical skills cannot be considered in isolation from other cognitive and academic abilities. Increasing evidence underscores the significant role of language skills in children's acquisition of early mathematical knowledge (Purpura et al., 2019; Erath et al., 2021). RVS, one of the foundational components of language development (Wilkins, 1972), refer to the words that an individual can understand and respond to, even if they are not able to produce these words themselves (Burger and Chong, 2011). RVS is recognized as a crucial tool in the development of mathematical concepts in children (Negen and Sarnecka, 2012).

Enhanced receptive vocabulary skills not only facilitates children's ability to acquire mathematical language through social interactions, but it also enriches their mathematical terminology and deepens their understanding of mathematical concepts (Klibanoff et al., 2006; Susperreguy and Davis-Kean, 2016). As noted by Barner et al. (2009), language skills play a decisive role in children's acquisition of cardinality knowledge for small numbers. Negen and Sarnecka (2012) further found that the larger the receptive vocabulary, the more profound children's understanding of the CP becomes. Similarly, Pixner et al. (2018) reported a significant correlation between children's vocabulary skills and their understanding of small number concepts.

Numerous studies have confirmed that children from higher SES backgrounds tend to outperform their peers from lower to middle SES backgrounds in terms of vocabulary skills (Fernald et al., 2013; Salminen et al., 2021; Xiao et al., 2020; Ma et al., 2024). These linguistic disparities can have significant implications for mathematical learning, particularly for children from low-SES families, for whom limited language development may pose a barrier to acquiring mathematical knowledge (Abedi and Lord, 2001). Further research has demonstrated that preschool children from middle-SES families perform better on tasks involving oral number combinations and mathematical story comprehension, possibly because they have more exposure to numerically relevant vocabulary (Jordan et al., 1992, 1994, 2006). Mastery of number-related vocabulary aids in the completion of mathematical tasks (Mix, 2008), and the lack of this vocabulary in low-SES preschoolers may place them at a disadvantage in their mathematical skill development (Bao, 2014).

Based on the above discussions, we propose the following hypothesis:

***Hypothesis 3 (H3)***: RVS partially mediates the relationship between SES and the development of children's CP.

### 1.4 The chain mediating effect of the Approximate Number System and Receptive Vocabulary Skills

Numerous studies have highlighted the critical role of ANS and RVS in children's numerical cognition development. Specifically, as the underlying mechanism for children's intuitive perception of quantities in early childhood, ANS enables children to perceive the differences in the quantity of objects, such as distinguishing which side of two piles of apples has more. This non-verbal sense of quantity based on ANS lays the foundation for children's subsequent digital cognitive development. However, for children to transform this intuitive perception of quantity into precise mathematical concepts, RVS plays a crucial mediating role (Spelke, 2003; Purpura and Logan, 2015; Barner, 2017; Spelke, 2017). As Spelke (2003) pointed out, language skills play a key mediating role in the process in which children refine their basic quantitative perception into accurate numerical representations. For example, when children learn to say “one apple” and “two apples,” they begin to use language symbols to gradually transform this non-verbal sense of quantity based on ANS into precise mathematical concepts (Barner, 2017).

As discussed in Sections 1.2 and 1.3, children from higher-SES families tend to have access to richer learning environments, creating more opportunities for interaction (Bradley and Corwyn, 2002; Evans, 2004). This environment helps children's ANS development, enhancing their sensitivity to quantity-related information and increasing their exposure to a broader vocabulary, thereby effectively boosting their RVS (Fuhs and McNeil, 2013; Golinkoff et al., 2019). Given these observations, the rich learning environment provided by a higher socioeconomic status (SES) not only benefits the cultivation of children's ANS, enabling them to perceive quantitative differences keenly but also creates favorable conditions for children to establish connections between this non-verbal quantitative intuition and precise numerical symbols through RVS.

Therefore, we propose the following hypothesis:

***Hypothesis 4 (H4):*** ANS and RVS mediate the relationship between SES and children's CP through a chain mediation effect.

### 1.5 The present study

The development of early mathematical skills, particularly mastery of the CP, plays a crucial role in determining later mathematical achievement and academic success. Among the many factors influencing children's CP development, SES, ANS, and RVS have attracted significant attention. However, the specific relationships and underlying mechanisms between these variables remain unclear. Therefore, we propose a chain mediation model to examine whether SES indirectly influences children's CP development through ANS and RVS. Empirical data will be collected from children across various socioeconomic backgrounds, assessing their sensitivity to ANS, level of receptive vocabulary, and understanding of CP. The SPSS PROCESS macro will be used to perform chain mediation effect analysis to test the proposed hypotheses. The results of the study will elucidate the intrinsic links and action pathways between SES, ANS, RVS, and CP.

**Figure 1:**
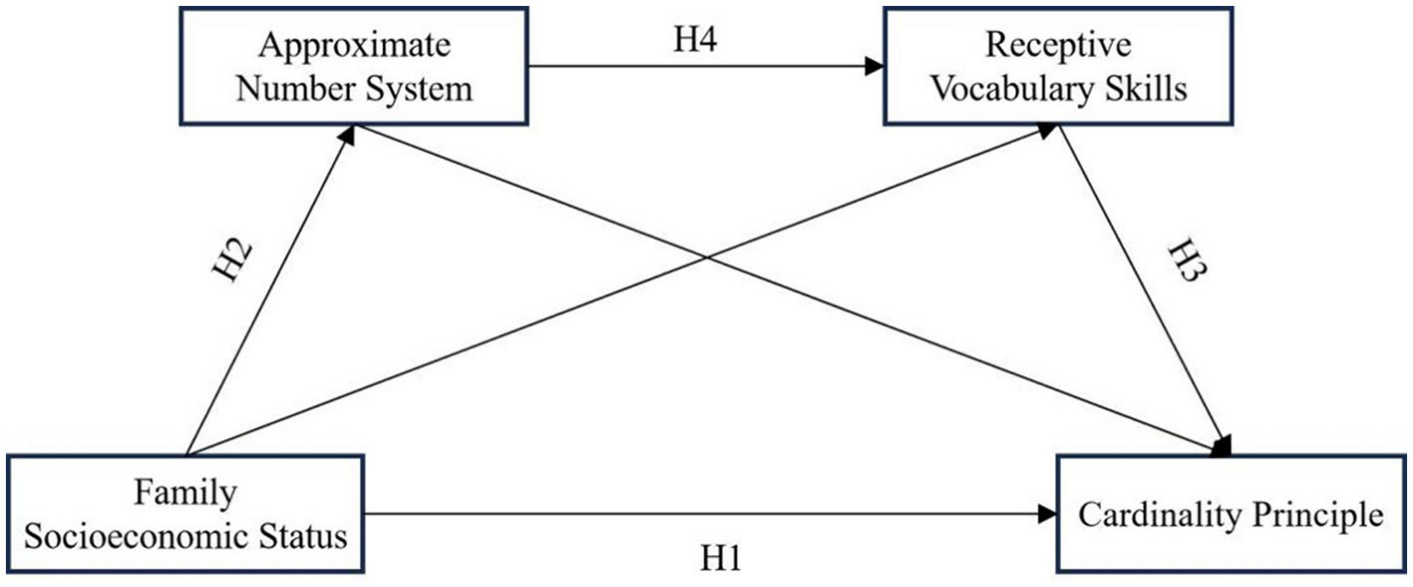
A visual representation of the proposed model.

A visual representation of the proposed model is shown in Figure 1.

## 2 Methods

### 2.1 Participants

The participants in this study were children from Urumqi City and Changji Prefecture in the Xinjiang Uygur Autonomous Region of China. Throughout the research process, we strictly adhered to ethical guidelines, ensuring that all children's guardians provided informed consent prior to participation. The inclusion criteria for participants were as follows: (1) children were able to understand and communicate in Mandarin Chinese; (2) participants did not have any intellectual or hearing impairment; and (3) children had normal vision or could achieve normal visual acuity through appropriate corrective measures. Additionally, the age range of participants was limited to children between the ages of 3 and 7 years.

Thanks to the active support of the management, teachers, and parents at the participating preschools, we successfully recruited 120 children and their families for the study. Despite challenges such as a flu outbreak, scheduling conflicts, and incomplete data collection, we ultimately collected a complete and high-quality dataset of 110 participants, which provided a solid foundation for our analysis. The sample consisted of an equal number of boys and girls (Mean age = 67.53 months, *SD* = 7.415 months). Table 1 provides detailed information on the participant characteristics.

**Table 1:** Sample characteristics.

**Variable**	**Categories**	***N***	**Percentage (%)**
Parental education level	Junior high school	7	6.4
	Junior college, Vocational high school, or High school technical school	16	14.5
	College	25	22.7
	Undergraduate	55	50.0
	Graduate or above	7	6.4
Average gross monthly household income	Less than 2,000 Yuan	7	6.4
	2,001–5,000 Yuan	26	23.6
	5,001–8,000 Yuan	38	34.5
	8,001–10,000 Yuan	20	18.2
	Above 10,000 Yuan	19	17.3
Parents' occupation	Temporary workers, unemployed, unskilled, and agricultural laborers	11	10.0
	Senior executives (managers) with professional technicians, supervisors	29	26.4
	Middle-level managers with technical staff, assistant professionals	52	47.3
	Manual laborers, self-employed, skilled workers	12	10.9
	General managers, transactional staff	6	5.5
Total		110	100

### 2.2 Procedure and measures

#### 2.2.1 Family Socioeconomic Status

The indicators for measuring SES include Parental Education Level, Parental Education Level and Parents' Occupation. Occupation, according to the research by Lin and Bian (1991), is classified into five levels from “Temporary workers, unemployed, unskilled, and agricultural laborers” to “General managers, transactional staff,” scored from 1 to 5 respectively; Parental Education Level is divided into five levels from “Junior High School” to “Graduate or Above,” scored from 1 to 5 respectively; Average Gross Monthly Household Income is classified into six levels from “Less than 2,000 Yuan” to “Above 10,000 Yuan,” scored from 1 to 5 respectively.

SES was measured using the method proposed by Ren (2010), which includes three key indicators: parental education level, occupational status, and family annual income. The evaluation procedure involved the following steps: first, each of these variables was assigned a quantitative score, which was then transformed into a Z-score to eliminate the effects of different measurement scales. Principal component analysis was then used to extract factor loadings (β1, β2, β3), representing the contribution of each variable to the first principal component. As a result, a principal factor with an eigenroot >1 was obtained, which explained 67.177% of the variance. It indicates that SES can be synthesized into one score, which is calculated by the formula:

SES = (β1^*^Z Parental Education + β2^*^Z Parental Occupation + β3^*^Z Annual Family Income)/ε*f*

where β1, β2, and β3 are the factor loadings for each variable, and ε*f* is the eigenvalue of the first principal component. Higher SES scores indicate higher socioeconomic status.

#### 2.2.2 Approximate Number System

To assess children's ANS, we used the Panamath test software developed by Halberda and Feigenson (2008). The core task of this tool involves a dot comparison task. Initially, a fixation point (“+”) is displayed to draw attention, followed by the simultaneous presentation of yellow and blue dot arrays triggered by a key press. The arrays are presented for a fixed duration of 2,128 ms, and participants are instructed to quickly and accurately judge the relative number of dots using the keyboard, with buttons pre-labeled by color. If a response is not made within the time limit, the task will automatically transition to a screen with a masking image and response box, requiring the participant to complete the response before continuing. The task consists of eight practice trials and 80 formal trials, with equal numbers of yellow and blue arrays. Upon completion, the software automatically calculates key metrics: reaction time, accuracy, and Weber fraction. The differences in the subjects' proficiency in operating the computer keyboard will impact reaction time. Moreover, some researchers have proposed that the Weber coefficient has limitations in accurately reflecting the acuity of approximate quantitative systems (Inglis and Gilmore, 2014; Peng et al., 2017). Therefore, in this study, only the accuracy rate was selected as an indicator to measure the acuity of the approximate quantitative system in young children. At present, the validity of this test method has been verified among young children in China (Peng et al., 2017).

#### 2.2.3 Receptive Vocabulary Skills

Children's receptive vocabulary skills were assessed using the Peabody Picture Vocabulary Test -Revised (PPVT-R), adapted by Guo et al. (2019). Prior to the official test, three practice sessions were conducted to ensure that all participants understood the task format and were familiar with the procedure. During the test, each target word was read aloud in standard Mandarin, and the child was asked to select the picture that best matched the meaning of the word from a set of images. Scoring followed these rules: one point was awarded for each correct answer, with no points given for incorrect answers. If the child made six or more errors in a consecutive sequence of eight items, the test was terminated early, and the score was recorded as the ceiling score. The final score was calculated by subtracting the number of errors from the ceiling score.

#### 2.2.4 Children's Cardinality Principle

Children's understanding of the CP was assessed using two tasks: the How Many task and the Give-n task. Although the “How Many” and “Give-n” tasks have potential differences in assessing children's understanding of the CP, given the characteristics of the current sample size and study design, we combined the results of these two tasks to obtain a composite CP score. This merging method allowed us to assess the overall performance of CP with more stable data while avoiding the reduced statistical power that would result from splitting the data.

***How many task***: In this task, children were presented with blocks and told: “The little bear is building a house with blocks, but it doesn't know how many blocks it has. You will be the teacher and help the bear count.” After two practice trials, three sets of formal tests were administered. In each set, children were asked to count the number of blocks in sets of 4, 7, and 9 blocks (Set 1), 13, 17, and 20 blocks (Set 2), and 22, 25, and 28 blocks (Set 3). Each test consisted of three trials, with a total of nine trials. A score of 1 was awarded for each correct response, and no score was given for incorrect responses. The total score for this task ranged from 0 to 9 points.

***Give-n task***: Before the test, present the blocks to the participants and explain the rules of the task. For example, instruct the child to take out a specified number of blocks out of 10, 20, and 30 blocks. Such as, “Please help the bear by taking out 6 blocks.” Then, conduct two practice trials to ensure understanding. Following the practice, proceed with three sets of formal test trials, each set containing three tasks. In the first set, children are asked to take out 4, 6, and 9 blocks from a set of 10 blocks. In the second set, they are to take out 12, 15, and 19 blocks from a set of 20 blocks. In the third set, they must take out 21, 24, and 28 blocks from a set of 30 blocks. This results in a total of nine tasks across the formal testing phase. Each correct response scores 1 point, while incorrect responses receive no points, with possible scores ranging from 0 to 9 points.

### 2.3 Covariates

Previous research has shown that both gender and age are significant predictors of the ANS, RVS, and the CP in children (Holloway and Ansari, 2009; Sekuler and Mierkiewicz, 1977; Sumit Kumar et al., 2022; Barsotti et al., 2023; Hornburg et al., 2018; Das et al., 2022). Therefore, in the current study, children's gender and age were included as covariates in the chain mediation analysis.

### 2.4 Data analysis

Data were analyzed using SPSS version 29.0. Descriptive statistics and correlation analyses were first conducted to assess the overall performance of the participants on SES, ANS, RVS, and CP, as well as to examine the relationships between these variables. Following this, we employed path analysis using PROCESS macro version 4.1 (Model 6) to test whether ANS and RVS mediate the relationship between SES and children's CP, controlling for children's gender and age. The significance of the mediation effects was evaluated using a bootstrap procedure with 5,000 resamples, with a 95% confidence interval (CI). A significant mediation effect was indicated if the bootstrap CI did not contain zero (Hayes, 2013).

## 3 Results

### 3.1 Correlation analysis

To explore the relationships between SES, ANS, RVS, and children's CP, we conducted descriptive statistics and correlation analyses. The results indicated significant positive correlations between SES (*r* = 0.443, *p* < 0.001), ANS (*r* = 0.447, *p* < 0.001), RVS (*r* = 0.560, *p* < 0.001), and CP. Additionally, SES (*r* = 0.446, *p* < 0.001) and ANS (*r* = 0.467, *p* < 0.001) were positively correlated with RVS, while SES (*r* = 0.304, *p* < 0.01) was also significantly positively correlated with ANS. These results provide preliminary support for the validity of our hypotheses. Means, standard deviations, and correlation coefficients for all variables are presented in Table 2.

**Table 2:** Descriptive statistics and correlation analysis.

**Variables**	***M***	***SD***	**1**	**2**	**3**	**4**	**5**	**6**
1. SES	0.000	2.999	1					
2. ANS	0.851	0.122	0.304^**^	1				
3. RVS	53.410	20.205	0.446^***^	0.467^***^	1			
4. HM	6.490	2.533	0.419^***^	0.355^***^	0.547^***^	1		
5. GN	5.860	2.862	0.410^***^	0.473^***^	0.503^***^	0.750^***^	1	
6. CP	6.177	2.524	0.443^***^	0.447^***^	0.560^***^	0.927^***^	0.943^***^	1

SES, Family Socioeconomic Status; ANS, Approximate Number System; RVS, Receptive Vocabulary Skills; HM, How Many; GN, Give-n; CP, Cardinality Principle.

** p <0.01,

*** p <0.001.

### 3.2 Analysis of the chain mediation model

To further investigate the relationships between SES, ANS, RVS, and children's CP, we conducted a chain mediation analysis using PROCESS macro (Model 6) developed by Hayes, with children's gender and age included as covariates. The results (see Table 3) indicated that SES significantly associated with ANS (β = 0.264, *p* < 0.01). Furthermore, both SES (β = 0.281, *p* < 0.01) and ANS (β = 0.338, *p* < 0.001) significantly associated with RVS. Additionally, SES (β = 0.164, *p* < 0.05), ANS (β = 0.191, *p* < 0.05), and RVS (β = 0.272, *p* < 0.01) significantly associated with CP. These results suggest that SES influences CP through both direct and indirect pathways via ANS and RVS.

**Table 3:** Regression analysis of the relationship of variables in the model.

**Regression equation**		**Overall fit index**			**Significance of regression coefficients**		
**Outcome**	**Predictor**	***R***	***R** ^2^*	***F***	β	***SE***	***t***
ANS	SES	0.345	0.119	4.777	0.264	0.388	2.773^**^
RVS	SES	0.602	0.362	14.883	0.281	0.569	3.328^**^
	ANS				0.338	0.138	4.064^***^
CP	SES	0.703	0.494	20.318	0.164	0.067	2.069^*^
	ANS				0.191	0.017	2.392^*^
	RVS				0.272	0.011	3.111^**^

SES, Family Socioeconomic Status; ANS, Approximate Number System; RVS, Receptive Vocabulary Skills; CP, Cardinality Principle.

* p < 0.05,

** p < 0.01,

*** p < 0.001.

**Figure 2:**
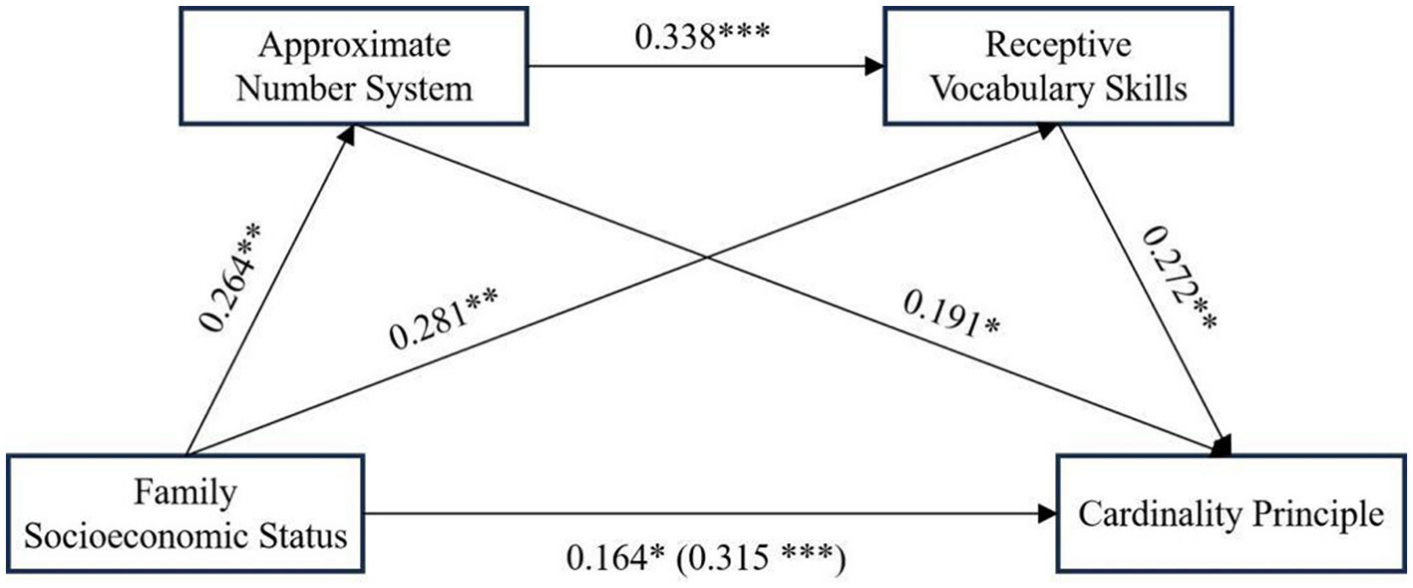
Chained mediation model diagram. Numbers in parentheses represent the total effects between SES and CP. *p < 0.05, **p < 0.01, ***p < 0.001.

Next, we used bootstrapping (5,000 resamples, 95% CI) to further test the chain mediation model. As shown in Table 4 and Figure 2, the total effect of SES on children's CP was 0.315. The mediation effects were decomposed into three distinct pathways: Path 1 (SES → ANS → CP) had an effect size of 0.051 [95% CI (0.0014, 0.3993)], Path 2 (SES → RVS → CP) had an effect size of 0.076 [95% CI (0.0249, 0.1373)], and Path 3 (SES → ANS → RVS → CP) had an effect size of 0.024 [95% CI (0.0053, 0.0581)]. The mediation effects accounted for 16.04%, 24.19%, and 7.67% of the total effect, respectively. The total indirect effect was 0.151, which accounted for 47.91% of the total effect of SES on children's CP. Since none of the 95% confidence intervals for these indirect effects contained zero, all mediation effects were statistically significant.

**Table 4:** Mediation effect of family socioeconomic status and early childhood cardinality principle.

**Cause Path**	**Effect**	**Boot SE**	**Boot LLCI**	**Boot ULCI**	**Effect ratio**
Total effect	0.315	0.068	0.132	0.399	100%
Direct effect	0.164	0.067	0.006	0.271	52.00%
Total indirect effect	0.151	0.043	0.072	0.238	47.91%
SES → ANS → CP	0.051	0.028	0.001	0.109	16.04%
SES → RVS → CP	0.076	0.029	0.025	0.137	24.19%
SES → ANS → RVS → CP	0.024	0.014	0.005	0.058	7.67%

SES, Family Socioeconomic Status; ANS, Approximate Number System; RVS, Receptive Vocabulary Skills; CP, Cardinality Principle.

## 4 Discussion

The present study examined the effect of SES on children's CP and the mediating role of ANS and RVS in this relationship in a series of tests with 110 children, and the results revealed several important findings: (1) SES, ANS, RVS, and CP were all significantly correlated with each other; (2) SES was significantly positively associated with children's performance on the CP task; (3) ANS and RVS partially mediated the relationship between SES and CP; and (4) ANS and RVS acted in a chained mediation role in the relationship between SES and CP. These findings highlight the complex pathways through which SES influences early mathematical understanding and demonstrate the critical roles of numerical cognition and language skills in shaping children's development of cardinality.

### 4.1 The relationship between family socioeconomic status and the cardinality principle

The results of this study indicate that SES has a significant positive effect on children's mastery of the CP, confirming Hypothesis 1. This finding is consistent with previous research (Rogelberg et al., 2021; Silver and Libertus, 2022; DePascale et al., 2023), which suggests that children from higher SES families tend to perform better in numerical domains. Conger and Donnellan (2007) proposed two influential models, the Family Investment Model and the Family Stress Model, to explain the “social causality” between SES and individual development. According to the Family Investment Model, parents from higher SES backgrounds have greater access to resources (Susperreguy et al., 2020) and are able to invest more material resources in their children's development (Conger and Dogan, 2007; Dearing and Taylor, 2007; Kaushal et al., 2011). Additionally, these parents are more likely to dedicate time and resources to engaging in enriching and enjoyable mathematical learning activities with their children (Roubinov and Boyce, 2017; Carneiro and Ginja, 2016), which effectively promotes the development of children's mathematical abilities.

In contrast, children from lower SES backgrounds typically have fewer opportunities to experience high-quality educational settings and demonstrate lower performance in early mathematics tasks and standardized math tests (Elliott and Bachman, 2018; Lyu et al., 2019; Short and McLean, 2023). From the perspective of the Family Stress Model, lower SES is associated with increased economic pressure, which can lead to family conflict, reduced parent-child interaction, and less academic guidance (Masarik and Conger, 2017). Economic stress may also limit the time parents can spend with their children, and their inability to invest in creating an optimal learning environment for their children can result in insufficient learning resources and a lack of attention to home-based learning activities (Conger et al., 2002). Such an environment restricts children's access to cognitive enrichment and learning guidance, further hindering their early academic development, including the mastery of concepts such as CP.

### 4.2 The mediating role of the Approximate Number System

The present study also found that the ANS partially mediates the relationship between SES and children's mastery of the CP, supporting Hypothesis 2. ANS is an innate numerical system that provides preschool children with an intuitive sense of quantity, allowing them to process object quantities before fully mastering symbolic number knowledge. This ability facilitates the understanding of the order and cardinality of numbers (Bethany et al., 2017; Chu et al., 2016). Previous studies have shown that the accuracy of the ANS is positively correlated with children's ability to master the CP; the greater the precision of the ANS, the better the children's understanding and mastery of CP (Huntley-Fenner and Cannon, 2000; Mussolin et al., 2012; van Marle et al., 2014; Wagner and Johnson, 2011).

Moreover, the level of family SES directly influences the quality of children's early mathematical experiences, and early math education plays a critical role in the development of ANS sensitivity (Pica et al., 2004; Fuhs and McNeil, 2013). Specifically, children from higher SES families are more likely to have access to high-quality educational resources and parent-child interactions, with more opportunities to engage in mathematical activities during these interactions (Bonny and Lourenco, 2013; Jiang and Wang, 2024). This enhances their ANS accuracy, which in turn facilitates a quicker mastery of the CP. In contrast, children from lower SES families, who lack effective mathematical guidance and resources (Fuhs and McNeil, 2013; Ramani and Siegler, 2008; Votruba-Drzal, 2003), may experience limited development of their ANS, which directly impacts their understanding and mastery of the CP. In addition, parents with a higher level of education tend to have higher expectations and more participation in their children's mathematics learning. This positive parent-child interaction and support further promoted the development of ANS (Keijer, 2021), thereby enhancing children's understanding of the CP. Consequently, the ANS acts as a bridge between SES and CP, partially explaining the differences in CP abilities observed in children from high and low SES families.

### 4.3 The mediating role of the Receptive Vocabulary Skills

Our findings also support Hypothesis 4, suggesting that RVS play a partial mediating role in the relationship between SES and children's mastery of the CP. This result aligns with previous research by Abedi and Lord (2001), which indicated that insufficient language development can be a barrier to children's early mathematical achievements, especially for those from low-income backgrounds. Vygotsky's theory posits that although thought and language originate from different processes, they ultimately converge, with language structures serving as the foundation for cognitive structures. This convergence limits cognitive development, particularly in the domain of mathematics, where language skills constrain mathematical thinking (Vygotsky, 1962). In this context, language skills are seen as a limiting factor in the development of mathematical abilities, such as understanding the CP.

Neurocognitive research has found that vocabulary skills and numerical cognition share common brain regions (Baldo and Dronkers, 2007; Arsalidou and Taylor, 2011), suggesting that children with stronger vocabulary skills tend to perform better in numerical tasks (Aunola et al., 2004). Furthermore, parents with a higher level of education tend to be able to provide a more language-rich environment (Bradley and Corwyn, 2002; Evans, 2004; Linver et al., 2002), where parents engage in more frequent verbal interactions with their children, use a more diverse vocabulary and complex sentence structures, and ask questions that stimulate conversation. This environment promotes the development of RVS (Golinkoff et al., 2019) and increases children's exposure to mathematical vocabulary, thereby laying a foundation for understanding the CP.

In contrast, children from lower SES families, due to resource constraints and differences in parental involvement, are likely to experience lower levels of language interaction and problem-solving activities, which restrict the development of their RVS (Bradley and Corwyn, 2002; Burchinal et al., 2008; Hoff, 2006). This limitation in RVS development, in turn, restricts their understanding of CP. Thus, RVS serves as a mediator between SES and CP, explaining part of the variation in CP mastery between children from different SES backgrounds.

### 4.4 The mediating effect of the Approximate Number System and Receptive Vocabulary Skills

An important finding of this study is that both the ANS and RVS play a chain-mediating role in the relationship between SES and children's mastery of the CP. ANS, an innate ability to approximate quantities, provides children with an initial, albeit vague, sense of number. As children's language skills, particularly their RVS, develop, they begin to link their intuitive sense of quantity with the vocabulary in language, transforming their vague number sense into a more precise representation of quantity (Piaget, 1977; Spelke, 2003). It is noteworthy that children's mathematical knowledge often develops through interactions that involve mathematical language (Klibanoff et al., 2006). For example, during everyday family conversations, parents and children might discuss shopping (e.g., “We need to buy 2 boxes of milk and 3 packs of cookies”), cooking (e.g., “We need 3 cups of flour to make a cake”), or organizing toys (e.g., “We have 5 stuffed animals and 4 toy cars—can you help me put them in different boxes?”). These language-based interactions help children connect their intuitive number sense to real-world contexts, thereby enhancing their understanding of CP (Barner, 2017; Spelke, 2017; Gibson et al., 2020; O'Rear and McNeil, 2019).

In higher SES environments, interactions between children and adults, especially those involving language and mathematics, tend to be more frequent (Bonny and Lourenco, 2013; Jiang and Wang, 2024; Li et al., 2023). These frequent interactions foster the development of a more precise ANS and further contribute to children's mastery of CP. However, parents engaged in physical labor or low-skilled jobs may find it challenging to provide their children with equally rich and high-quality learning stimuli at home due to the limitations of their working hours and energy and the influence of their educational background. This may lead to relatively lagging children in the development of ANS, RVS, and CP. Thus, both ANS and RVS play a critical, interconnected role in mediating the effects of SES on children's understanding of mathematical concepts.

## 5 Contributions, limitations, and future directions

This study aims to analyze how SES influences children's mastery of the CP, while also exploring the roles of ANS and RVS in this process. The results demonstrate that SES has a significant positive predictive effect on children's performance in CP tasks. Both ANS and RVS act as partial mediators in the relationship between SES and CP, with ANS and RVS together exerting a chain-mediating effect. This study offers several important contributions:

***Theoretical contributions***: This study makes an important theoretical contribution by empirically validating the model of SES via ANS and RVS chain mediation influencing children's CP development. Distinguishing from previous studies that have explored these factors in isolation or in parallel, this chain-mediated effect elucidates the specific mechanistic steps by which SES influences early mathematical development, integrating mathematical cognition and language development into a unified framework, and demonstrating their interdependence in basic mathematical learning. This study provides new theoretical perspectives and empirical evidence for understanding the link between SES and CP in a more refined way than a single mediator or direct influence model, and lays a stronger theoretical foundation for in-depth investigations of the complex interactions between environmental factors and domain-specific cognitive abilities in early mathematical cognitive development.

***Educational implications***: These findings are relevant to educational practice because educators can use this knowledge to design targeted educational interventions. First, the chain pathway (SES → ANS → RVS → CP) identified by the study suggests that it is important to focus on both basic ANS and RVS development in preschool and the lower primary grades. An intervention that combines non-symbolic quantity discrimination training with vocabulary development training is more effective in enhancing children's mathematical competence than a single domain intervention; Second, the results emphasize the importance of early screening and the need to identify potential deficits in ANS and RVS in children from low SES families as early as possible in order to provide timely, targeted support before these children formally learn math concepts such as CP, and to avoid the accumulation and widening of early gaps; Third, given the correlation between ANS and RVS, early education should integrate math's activities with language-rich experiences, such as embedding number play, comparison and counting activities in storytelling, discussion and communication, and descriptive play. This educational model can simultaneously promote children's ANS, RVS, and CP development; finally, while cognitive interventions are crucial, the findings also highlight the profound impact of socioeconomic inequality on children's early cognitive development. This finding provides a strong basis for the development of social and educational policies aimed at reducing socioeconomic disparities and ensuring that all children have access to high-quality early learning environments that are rich in numeracy and language stimulation.

Despite its contributions, the study has several limitations:

***Sample size and generalizability***: The sample size of this study was relatively small, and the sample size of 110 participants may not have been sufficiently powered to detect small mediating effects. At the same time, this study did not fully account for the diversity of children's backgrounds, such as regional, cultural, or ethnic differences, which may have influenced the results. Future studies should aim to increase sample sizes and include children from diverse geographic, cultural, and ethnic backgrounds to enhance the detection of smaller mediating effects and provide more robust evidence for the proposed pathways, ensuring that findings are more generally applicable.

***Cross-sectional design***: This study used a cross-sectional design, which limits our understanding of the dynamic nature of the relationships between SES, ANS, RVS, and CP over time. A longitudinal study would allow for a more comprehensive examination of how these factors interact and evolve throughout different stages of children's development, providing insights into long-term developmental trends.

***Methods:*** This study combined scores from the “How Many” and “Give-n” tasks into a single integrated CP score to assess children's understanding of the CP. Although these two tasks are highly correlated when assessing CP, O'Rear et al. (2024) found that children's performance on these two tasks may differ, suggesting that they may capture different aspects of numerical cognition. Therefore, combining these two tasks may have limited our meticulous analysis of mediating effects to fully reveal the specific roles of ANS and RVS in different tasks. Future studies should further explore the independence of “How Many” and “Give-n” tasks with ANS and RVS, respectively, in order to understand the development of children's mathematics cognition more comprehensively.

***Uncontrolled variables***: Although this study focused on SES, ANS, and RVS, other factors—such as children's individual physiological and psychological traits, family emotional climate, and community environment—could also influence CP development. These uncontrolled variables may have affected the results and led to some limitations in the precision of the conclusions. Future studies should explore additional factors, such as parental beliefs, home numeracy, peer relationships, and early childhood education quality, to examine their interactions with SES, ANS, and RVS in shaping children's mathematical development.

## 6 Conclusion

This study investigates the impact of SES on children's mastery of CP and explores the mediating roles of ANS and RVS. The results from 110 children show that SES has a significant positive effect on children's CP, with ANS and RVS each playing partial mediating roles, and together, they exert a chain-mediating effect between SES and CP. These findings enhance our understanding of the relationship between SES and children's CP, expand the theoretical framework in this field, and highlight the importance of ANS and RVS in this process. Moreover, the study sheds light on the interconnected pathways between SES, ANS, RVS, and CP, filling gaps in previous research and offering new perspectives on the relationship between SES and children's mathematical ability development. The findings also provide empirical evidence that can inform early math education and efforts to promote educational equity.

## Data Availability

The original contributions presented in the study are included in the article/supplementary material, further inquiries can be directed to the corresponding author.
